# Supplementary material for: Patterns of Intron Gain and Loss in Fungi
Source: PLoS Biol. 2004 Nov 30;2(12):e422. doi: 10.1371/journal.pbio.0020422 (PMC532390; doi:10.1371/journal.pbio.0020422)
Supplement: Table S1 — Also available at http://genes.mit.edu/NielsenEtAl/. (4.3 MB ZIP). [file pbio.0020422.st001.zip › NielsenEtAl/html/885.html]

AN5718.1.NCU08959.1.MG06186.1.FG10367.1


```
 CLUSTAL W (1.82) Multiple Sequence Alignments - Introns Inserted


Sequence 1: NCU08959.1	397 aa
Sequence 2: MG06186.1	471 aa
Sequence 3: FG10367.1	454 aa
Sequence 4: AN5718.1	440 aa
Alignment Length: 480 aa
Number Identitical Residues: 169 aa
Alignment Score (without introns) 9248


MG06186.1 	MDTPALPAPRDAREKEILERLTATRDQLLLLKQDRAHYIRSQDVMVLYDQVIENVRQLNE
NCU08959.1	METPAMPMPKDEREQAILDKLSAIRDQLLLLKRDRTKYIRTQDVIVHYDQVVEQVKELNE
FG10367.1 	MDTPAIPMPRDPREQDILQKLTMIRDRLLLLKQDRTNYIRTQDVMPLFDETMDQVKDLTV
AN5718.1  	MESPAIQTPLDPKEQPILESVLRIRDALYLLKQDKSSYIRSRDVLPLYEEVTKEIEKLNA
          	*::**:  * * :*: **: :   ** * ***:*:: ***::**:  :::. .::..*. 

MG06186.1 	IRHGE--HGGETQL1DKVLESCFQLISLFFMTIGRTSEAPAA~YALTSTIR~RLLDHLAE
NCU08959.1	IRKGE--KVTETR-~---------------------------~--------~--------
FG10367.1 	VRAETG-LKEENRL1DKILESCFQLLSLFYLTIGRNNEAPAT~YAMTSTVK~RLLDHLNE
AN5718.1  	VRKDQNRRLAHNRL1DYVLDDCFQLISLLFLTVGRNNEAPAV2YSLAATVQ0RLLDHLEE
          	:*   .    ..:  .   ... .  :    : . ...:.:   : :::      .   .

MG06186.1 	AELFSA~KDLESMSETLQKISGVVRST------ESRHSPYLVELLSNRLELCQRNLENLQ
NCU08959.1	-----A1KDLNSIAHTLSQLDKIMTASD-----PSAHSPYIVELLANRLELCHSSLDNLK
FG10367.1 	AELYSA~KDLGSIKTTLEDLSKNIREAASDPSPDKRHPPYMLTLLENRVELCNSTLSRLQ
AN5718.1  	AGFYSA~KDLSSITTTLAGMRETLDRG------KEAHSPALLTLLESRLDKCQIKLEKLQ
          	:   :* *** *:  **  :   :          . *.* :: ** .*:: *: .*..*:

MG06186.1 	RRLDHLGPPLPAIHEKLISILRSMSLANTRSK0FSSSEVVKLQAELKEIDARRVDGNFVD
NCU08959.1	KRVDELQDPLQSMHERLISILRSMSLANTKAK0FSASEVQRLQAQVKEIDESRVDGNFVG
FG10367.1 	KRLDKLPDYLLESHEKLISILRSISLANTKSK0FSTSEVKKLRNQILEIGEKHSNGTFTA
AN5718.1  	SELTKLSPELASTHETLVSVLRSTAAVNTRSK0FSAAEVNGLRDQLKRIEDSMKDGNFVD
          	 .: .*   *   ** *:*:*** : .**::* **::**  *: :: .*     :*.*. 

MG06186.1 	ENGNVARGSDAVATLLKRCLEWTEIVLDR2KGRVPESFKNIYDVLVSIRNKLDSLSLTQA
NCU08959.1	PDGQVLRGSERVSRLVDVCLRWSDIVLER2KGVIPEAFKARHEQLVSIRNELEKLSITQA
FG10367.1 	EDGAVEEGGEVVRDLYNRCVRWSDLVLER2QGEVADQWRPIYDTLIRIRNDLEKLSLTQA
AN5718.1  	ANGNVLDNQEEVKLLLQRCWRWTEIVLER2EGKIDERFREQYERLLDIRNQLDRLSVTQA
          	 :* *  . : *  * . * .*:::**:* :* : : ::  :: *: ***.*: **:***

MG06186.1 	WSLRETDLYDFQRTLDKIDERR-VNGNFIDEEGRPAELYVQR0TLLYLIRRSYGLIYSLM
NCU08959.1	WSLRETDLYDYQRKLDKIDESR-VDGNWLDDEGKPAELYVQR~TLLYLIRRSYGYIYYLM
FG10367.1 	WSLRETDLYDFQRQLDRIDESRGDNGNWVDDRGRSADLWTQR~TFLYLIRRSYAYIYSFM
AN5718.1  	WSLRETDLFVYQRKLDRIDEAR-VNGNFVDAEGKPADLHAQR0TLLYLIRRSYAYIYALL
          	********: :** **:*** *  :**::* .*:.*:* .** *:********. ** ::

MG06186.1 	ILSEPVSEALIPIYNQLQTLKRILIEVKDSGGVSSVRELYPYSMK0LNMIDNMRVDGRFM
NCU08959.1	ISSEPVSEALLPIYNQLQTLKRCLIEVKDSGGVSSVRELYPYSMK0LNSIDNMRVDGKFM
FG10367.1 	LASEPVSEALLPVYNQLQTLKRCLIEVKNNGGVSSVRELYPYSMK0LNSLDNMKVDGKFV
AN5718.1  	ISSEPVSEALLPVYNQLQTLRRCLLEVKESGGVSNSRELYPYSMK0LNSIDNMRVDGKFY
          	: ********:*:*******:* *:***:.****. ********* ** :***:***:* 

MG06186.1 	HGDDIPEGQGALNELLADCYELAYELRVVAEAKAEETQAEDEPRPEDEPGTNGGSTAGSR
NCU08959.1	VGGDIPEGQGSVSALLAECYDLSYELKVMAEEMEEKEKDSSS------------------
FG10367.1 	VNGDIPEGQGSVTELLHECFDLNYELRVAAEEVIENNANSNEA-----------------
AN5718.1  	IGNDIPEGQGGVNALLAECYDLVWELRAAVADDKE-------------------------
          	 ..*******.:. ** :*::* :**:. .    *                         

MG06186.1 	ITRDEVLT
NCU08959.1	--------
FG10367.1 	--------
AN5718.1  	--------
          	
```
